# Oral anticoagulant reversal and mortality in trauma patients: a multicentre propensity score–matched cohort study

**DOI:** 10.1016/j.eclinm.2025.103577

**Published:** 2025-10-16

**Authors:** Elodie Lang, Marion Gautier, Jean-Luc Hanouz, Fanny Vardon, Vincent Legros, Gary Duclos, Florent Hericher, Gerard Audibert, Delphine Huet-Garrigue, Paer-Sélim Abback, Benjamin Popoff, Olivier Duranteau, Samy Figueiredo, Pierre-Antoine Allain, Thomas Botrel, Jean Pasqueron, Anne Godier, Traumabase® Group

**Affiliations:** a Department of Anesthesiology and Critical Care, AP-HP, Hôpital Européen Georges Pompidou, Paris, France; b Department of Anesthesia and Critical Care, Caen University Hospital, and Université Caen Normandie, INSERM UMR-S 12373, Caen, France; c Pole Anesthésie-Réanimation CHU Toulouse, INSERM U1297 équipe 11 LIPSIPLAT, Toulouse, France; d Department of Anesthesiology and Critical Care, Reims University Hospital, Université de Reims Champagne-Ardenne, UR 3797 VieFra, Reims, France; e Department of Anaesthesiology and Intensive Care, Hôpital Nord, Assistance Publique - Hôpitaux de Marseille, Marseille, France; f Département of Anesthesiology, Hôpital de Hautepierre-Hôpitaux Universitaires de Strasbourg, Université de Strasbourg, 67098, Strasbourg, France; g Université de Lorraine, CHRU-Nancy, Department of Anesthesiology and Critical Care, F-54000, Nancy, France; h Department of Anesthesia and Critical Care, CHU de Lille, Lille, France; i Department of Anesthesiology and Critical Care, Tours University Hospital, 37000, Tours, France; j CHU Rouen, Department of Anesthesiology, Critical Care and Perioperative Medicine, F-76000, Rouen, France; k Intensive Care Unit, HNIA Percy, Clamart, France; l Service Anesthésie-Réanimation Médecine Péri Opératoire, Assistance Publique – Hôpitaux de Paris, Hôpital de Bicêtre, Le Kremlin Bicêtre, 94275, France; Équipe DYNAMIC Dysfonction d'organes et Microcirculation (UMR-S999), Université Paris-Saclay, France; m Department of Anesthesiology and Critical Care, AP-HP, Beaujon Hospital, Clichy, France; n Department of Anesthesiology and Critical Care, Sorbonne University, GRC 29, AP-HP, DMU DREAM, Pitié-Salpêtrière Hospital, 43-87 Bd de l'Hôpital, 75013, Paris, France; o Department of Anesthesiology and Critical Care, AP-HP, Henri Mondor Hospital, Créteil, France; p Université Paris Cité, INSERM the Paris Cardiovascular Research Center, Team Endotheliopathy and Hemostasis Disorders Paris, France

**Keywords:** Trauma, Oral anticoagulant, Reversal, Bleeding, Mortality, Thrombosis

## Abstract

**Background:**

Oral anticoagulant (OAC) therapy increases bleeding risk but its impact on trauma outcomes and the benefit of reversal remains uncertain. This study aimed to evaluate 1/the effect of preinjury OAC therapy on trauma mortality and 2/the protective role of OAC reversal and its associated thrombotic risk.

**Methods:**

We conducted an observational study using a prospective multicenter trauma registry between January 2012 and December 2023. OAC-treated patients were matched with non-OAC-treated patients using a propensity score. Univariable and multivariable logistic regressions assessed associations between OAC therapy and day 1 and day 7 mortality. The effect of guideline-concordant OAC reversal was evaluated. Thrombotic complications were recorded.

**Findings:**

Of the 27,426 trauma patients, 3% were OAC-treated. They were older, had more comorbidities, and experienced higher mortality. After matching (n = 2196), OAC therapy remained independently associated with increased mortality (day 1: OR 2·21, 95% CI [1·41–3·43]; day 7: OR 2·06, [1·41–3·00]), with greater risk from vitamin K antagonists (VKA) than direct oral anticoagulants (DOAC). Guideline-concordant OAC reversal, achieved only in 21% of cases, independently reduced mortality at day 1 (OR 0·10, 95% CI [0·03–0·31], p < 0·01) and day 7 (OR 0·51, 95% CI [0·22–0·97], p < 0·01). No significant association was found between reversal and thrombotic complications.

**Interpretation:**

Preinjury OAC therapy substantially increased trauma mortality, particularly with VKA. Guideline-concordant reversal was associated with reduced mortality in both VKA- and DOAC-treated patients without excess thrombotic risk but remains underused. These findings emphasise the urgent need for systematic implementation of reversal strategies in OAC-treated trauma patients.

**Funding:**

The Traumabase registry is funded by several Regional Health Agencies (Agences Régionales de Santé, ARS): ARS Île-de-France, ARS Occitanie, ARS Grand Est, 10.13039/501100014160ARS Hauts-de-France, and ARS Auvergne-Rhône-Alpes. The registry is also funded by the French Road Safety Observatory—Road Safety Delegation Service (Observatoire National Interministériel de la Sécurité Routière—Délégation à la Sécurité Routière).

**Research in context**

**Evidence before this study**

We searched PubMed and Embase from January 1, 2010, to January 1, 2024, using the terms “trauma”, “oral anticoagulants”, “vitamin K antagonists”, “warfarin”, “DOAC”, “reversal”, and “mortality”. Prior studies suggest that oral anticoagulants (OAC), particularly vitamin K antagonists (VKA), may worsen trauma outcomes. However, the benefit of anticoagulant reversal remains uncertain. Most data come from non-trauma populations and focus on laboratory endpoints or hematoma expansion rather than mortality. In trauma patients, evidence is sparse, observational, and conflicting. No randomised trial has demonstrated that OAC reversal improves survival.

**Added value of this study**

ARIANE is the first large multicentre study to assess the impact of OAC therapy and its reversal on mortality of trauma patients. Using a national prospective trauma registry supplemented with detailed retrospective data on anticoagulation management from 17 level I trauma centres, we found that VKA use and DOAC were independently associated with increased early mortality. Importantly, guideline-concordant reversal of either VKA or DOAC therapy significantly reduced day-1 and day-7 mortality, without increasing thrombotic events. These associations remained robust after propensity score matching and multivariable adjustment. To our knowledge, this is the first study to demonstrate the survival benefits of OAC reversal in trauma patients using real-world data.

**Implications of all the available evidence**

As the use of OACs increases, trauma care must adapt. Our findings support early, protocol-driven reversal of OAC in bleeding trauma patients. They emphasise the importance of adopting tailored, agent-specific reversal strategies, and advocate the implementation of structured protocols in trauma networks.

## Introduction

Post-traumatic bleeding is the leading cause of preventable death in injured patients, with one-third exhibiting signs of coagulopathy upon hospital admission.^1^, ^2^, ^3^, ^4^ Trauma-associated coagulopathy is a complex, multifactorial failure of haemostasis characterised by endothelial dysfunction, systemic anticoagulation, hyperfibrinolysis and hypofibrinogenemia. It is further exacerbated by shock, acidosis, hypocalcemia, hypothermia, and dilution from fluid administration. Patient-specific factors modulate the coagulopathy profile, such as age, comorbidities and medications, particularly oral anticoagulants (OAC) including vitamin K antagonists (VKA) and direct oral anticoagulants (DOAC).

As OACs increase the risk of bleeding, international guidelines advise their rapid reversal in the bleeding patient considering either prothrombin complex concentrates (PCC) to reverse VKA or DOAC, or specific reversal agents, such as andexanet alfa and idarucizumab for anti-Xa DOAC and dabigatran respectively.^1^^,^^5^^,^^6^ However, the low grade of these recommendations highlights the limited evidence that reversal improves clinical outcomes. No randomized controlled trial (RCT) has ever demonstrated that VKA or DOAC reversal reduces mortality in any population.

In trauma patients, the benefit of anticoagulant reversal has been poorly evaluated and the few available studies are observational, with conflicting results.^7^, ^8^, ^9^ Even the impact on mortality of pre-injury OAC therapy, especially DOACs, remains unclear. As the prevalence of anticoagulation is increasing in the general population, including trauma patients, it is essential to better define its management and clarify the role of reversal in trauma patients.

The aims of our study were to assess 1/the prevalence of pre-injury OAC therapy in a large cohort of trauma patients admitted to level I trauma centres, 2/the effect of OAC on trauma mortality, 3/the potential protective role of OAC reversal on mortality and 4/the association between reversal and thrombotic events.

## Methods

### Study design and trauma centres

This was a retrospective study conducted on patients whose data were prospectively collected between January 2012 and December 2023 in the Traumabase® (www.traumabase.eu), a French major trauma registry. Patients included in the Traumabase® are suspected of severe trauma from the scene and admitted to the participating trauma centres (n = 26).

### Ethics

The Traumabase® is in accordance with all requirements from the Advisory Committee for the processing of research information in the field of health (CCTIRS), the French National Commission on Computing and Liberty (CNIL, authorization number 911461) and meets the requirements of the local and national ethics committee (Comité de Protection des Personnes, Paris VI- Société Française d’Anesthésie-Réanimation). The structure of the database integrates algorithms for consistency and coherence and the data monitoring is performed by a central administrator. As this was a retrospective study, the informed consent was waived.

### Population

First analysis (Global cohort): we included all trauma patients recorded in the TraumaBase® registry by the participating centres during the predefined period. Patients under 18 years of age or those managed after secondary transfer were excluded. The registry dataset collected demographic information, trauma characteristics and management, laboratory values and outcomes. The Injury Severity Score (ISS) was calculated once the whole-body injury assessment was completed. The Trauma and Injury Severity Score (TRISS) was used to estimate the expected probability of survival.

Second analysis (ARIANE Cohort): Due to the limited granularity of anticoagulant data in the TraumaBase® registry —restricted to age, sex, BMI, American Society of Anesthesiologists (ASA) Physical Status Classification, antithrombotic therapy (yes/no)—, 17 out of the 26 Traumabase® centres retrospectively collected additional data for the purpose of this study. This supplemental data included information on preinjury OAC therapy (type of agent, indication and reversal strategies such as four-factor PCC, idarucizumab, andexanet alfa (available only in research settings), or recombinant activated factor VII), as well as bleeding and thrombotic complications. Significant bleeding was defined as the transfusion of at least 1 packed red blood cell (RBC) within the first 6 h of management. Traumatic brain injury (TBI) was defined as intracranial bleeding on the admission CT scan. OAC-treated patients were matched with non-OAC-treated patients using a propensity score approach (see below). Together, these patients comprised the ARIANE cohort.

OAC reversal was assessed within the first 24 h of hospital admission and classified into 3 groups according to French guidelines^6^ as followed.

**Table:** 

	VKA	anti-Xa DOAC	Dabigatran
Guideline-concordant reversal	PCC dose ≥25 UI/kg	PCC dose ≥50 UI/kg for intracranial haemorrhage and ≥25 UI/kg for other bleeding injury or andexanet alfa (available only in research settings)	PCC dose ≥50 UI/kg for intracranial haemorrhage and ≥25 UI/kg for other bleeding injury or idarucizumab 5 g
Incomplete reversal	PCC dose <25 UI/kg	PCC dose <50 UI/kg for intracranial haemorrhage and <25 UI/kg for other bleeding injury	
No reversal	no PCC given		

### Study outcomes

The objectives of the study were:

- To define the prevalence of OAC-treated patients and their mortality in the global cohort of trauma patients admitted in the 17 level I trauma centres.
- To assess the specific effect of OAC therapy on mortality in the ARIANE cohort that included OAC-treated patients and matched non-OAC-treated patients.
- To assess the potential protective role of OAC reversal on all-cause 1-day and 7-day mortality in the ARIANE cohort.
- To describe anticoagulant resumption, bleeding and thrombotic complications in OAC-treated patients who were hospitalised in the ICU for more than 48 h.

### Statistical analysis

The report follows the Strobe guidelines.^10^ Continuous variables were expressed as median [interquartile ranges] and compared using the Wilcoxon test. Categorical variables were expressed as percentages and analysed using Fisher's exact test. A p-value <0·05 was considered statistically significant. Missing data were handled using multiple imputation by chained equations (MICE). The proportion of missing data was low (<5%) for all variables included in the analysis. Twenty imputations were performed to ensure robust estimates. Predictive mean matching was used for continuous variables, logistic regression for binary variables, and polytomous regression for categorical variables. Propensity scores were estimated within each imputed dataset, and all analyses were pooled using Rubin's rules.

To assess whether OAC therapy was an independent risk factor for all-cause 1-day and 7-day mortality, we first matched OAC-treated patients and non-OAC-treated patients using a propensity score (ARIANE cohort). Variables considered for matching included age, sex, injury mechanism, and year of injury. Propensity scores were estimated using logistic regression. A 1:4 nearest-neighbor matching without replacement was performed using a caliper of 0·05 on the propensity score scale. Covariate balance between matched groups was assessed using absolute standardized mean differences (SMDs), with a cutoff value of <0·1 considered indicative of adequate balance. The C-statistic of the propensity score model was 0·89 (95% CI 0·88–0·91, indicating good discrimination. The association between OAC therapy and mortality at day 1 and day 7 was assessed using univariable and multivariable logistic regression. Univariable analyses identified factors for inclusion in multivariable analyses using a p-value <0·20 as the threshold. A stepwise Akaike Information Criterion procedure was performed to select a reduced set of predictor variables for building the best-performing multiple logistic regression model.

To assess whether OAC reversal was an independent protective factor for all-cause 1-day and 7-day mortality, we performed univariable and multivariable logistic regression in the population of OAC-treated patients. The criteria for identifying factors associated with mortality were the same as those previously described. We then assessed the association between the quality of anticoagulant reversal and in-hospital mortality according to the type of anticoagulant using Cox proportional hazards models in the ARIANE cohort. Time zero was hospital admission, and patients were followed until in-hospital death or discharge. The proportional hazards assumption was tested using Schoenfeld residuals and log-minus-log plots. Statistical analyses were carried out using R® 4·2·2 software (*R Foundation for Statistical Computing, Vienna, Austria*).

### Role of funding source

The Traumabase registry is funded by several Regional Health Agencies (Agences Régionales de Santé, ARS): ARS Île-de-France, ARS Occitanie, ARS Grand Est, ARS Hauts-de-France, and ARS Auvergne-Rhône-Alpes. The registry is also funded by the French Road Safety Observatory—Road Safety Delegation Service (Observatoire National Interministériel de la Sécurité Routière—Délégation à la Sécurité Routière). The funders had no role in study design, data collection, data analysis, decision to publish, or preparation of the manuscript. Authors were not precluded from accessing data in the study and accept responsibility for the decision to submit the manuscript for publication.

## Results

### OAC-treated patients: prevalence and mortality

**Fig. 1:**
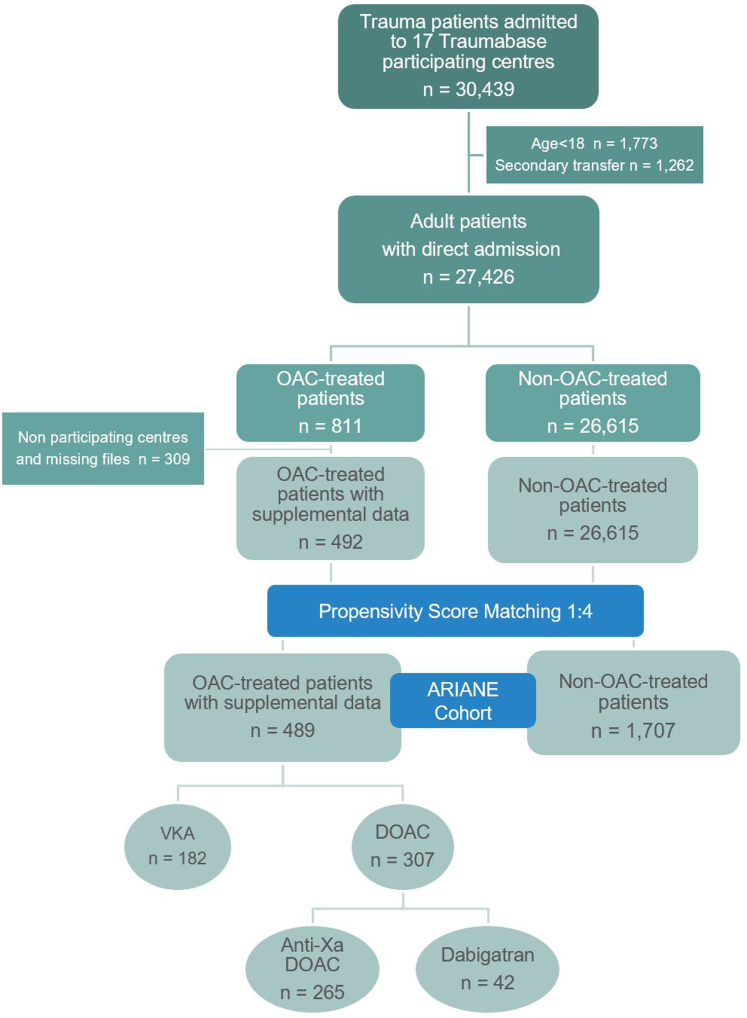
*Flowchart of the study*.

During the study period, 30,439 patients were admitted to the participating trauma centres (Fig. 1). After excluding those who did not meet the defined criteria, 27,426 patients remained for the first analysis (global cohort), 811 of whom (3%) had received OAC therapy prior to injury (Table 1).

**Table 1:** Characteristics of each subpopulation of the global cohort.

	Global cohort n = 27,426	OAC-treated patients n = 811	Non-OAC treated patients n = 26,615	SMD	p
Age (years)	38 [26–54]	75 [65–84]	37 [26–52]	1·93	<0·01
Male	21,566 (78·6%)	582 (71·8%)	20,984 (78·8%)	0·16	0·01
ASA score	1 [1–2]	2 [2–3]	1 [1–2]	1·69	<0·01
BMI (kg/m2)	24·6 [22·2–27·5]	26·2 [23·4–29·4]	24·5 [22·2–27·3]	0·34	<0·01
Antiplatelet agent	1315 (4·8%)	152 (18·9%)	1163 (4·4%)	0·46	<0·01
Penetrating trauma	3407 (12·4%)	83 (10·2%)	3324 (12·5%)	0·23	0·77
Total ISS score	13 [5–22]	16 [8–25]	13 [5–22]	0·16	<0·01
Significant bleeding	2495 (9·1%)	87 (10·7%)	2408 (9·1%)	0·22	0·14
Traumatic brain injury	5419 (21·3%)	225 (31·7%)	5194 (21·0%)	0·24	<0·01
TRISS score	0·02 [0·01–0·07]	0·07 [0·03–0·35]	0·01 [0·01–0·06]	0·43	<0·01
1-day mortality	1286 (4·7%)	115 (14·2%)	1171 (4·4%)	0·34	<0·01
7-day mortality	2170 (7·9%)	199 (24·6%)	1971 (7·4%)	0·48	<0·01
1-day mortality in ISS >15 patients	1085 (10·3%)	96 (28·4%)	989 (9·7%)	0·41	<0·01
7-day mortality in ISS >15 patients	1836 (17·4%)	157 (46·4%)	1669 (16·4)	0·32	<0·01

ASA score, American Society of Anesthesiologists score; BMI, Body Mass Index; TRISS, Trauma and Injury Severity Score; ISS, Injury Severity Score.

Mortality at day 1 from admission was nearly three times higher in OAC-treated patients than in non-OAC-treated patients, both in the overall cohort and among patients with severe trauma (ISS >15): 14·2% vs 4·4% and 28·4% vs 9·7%, respectively. Mortality at day 7 was also three times higher in OAC-treated patients, reaching 46% in those with severe trauma.

However, the pre-injury characteristics of the two populations differed significantly: OAC-treated patients were much older and had more comorbidities as indicated by a higher ASA classification, higher body weight and a greater frequency of antiplatelet therapy (Table 1). They also had more severe trauma, as indicated by a higher ISS score, with a greater incidence of TBI.

### Effects of OAC therapy on mortality

After propensity score matching (Figure S1, Supplementary Data), the ARIANE cohort included 2196 patients (Table 2): 1707 patients without pre-injury OAC therapy and 489 OAC-treated patients (182 (37·2%) on VKAs, 265 (54·2%) on anti-Xa DOACs and 42 (8·6%) on dabigatran). The proportion of DOAC use increased over time, from 0% in 2012 to 2·7% in 2022 (Figure S2, Supplementary Data). The primary indication for OAC therapy was atrial fibrillation (n = 327, 66·9%), followed by venous thromboembolism (n = 81, 16·5%) and mechanical heart valve (n = 10, 2·0%). In 71 patients, the indication was not documented. Of the 58% of OAC-treated patients for whom anticoagulant level was measured upon admission, 20% and 21% of those treated with VKA and DOAC, respectively, were not actually anticoagulated.

**Table 2:** Characteristics of each subpopulation of the ARIANE cohort.

	OAC-treated patients n = 489	Matched non-OAC-treated patients n = 1707	p
Patient and Injury Characteristics			
Age (years)	74·0 [63·0–82·0]	73·0 [62·0–81·0]	0·16
Male	364 (74·4%)	1290 (75·6%)	0·43
Year of injury	2019 [2018–2021]	2019 [2017–2021]	0·62
ASA score	3 [2–3]	2 [1–2]	<0·01
BMI (kg/m^2^)	26·0 [23·0–29·0]	24·0 [22·0–27·0]	<0·01
Antiplatelet agent	86 (17·6%)	385 (22·5%)	0·03
Penetrating trauma	54 (11·0%)	209 (12·2%)	0·83
Total ISS score	16 [9–25]	16 [9–25]	0·25
TRISS score	0·09 [0·03–0·37]	0·07 [0·03–0·33]	0·27
Prehospital Phase			
Prehospital GCS	15 [8–15]	15 [9–15]	0·32
Prehospital intubation	138 (28·2%)	478 (28·0%)	0·43
Shock Index	0·63 [0·50–0·80]	0·63 [0·50–0·78]	0·96
Prehospital MAP (mmHg)	97 [78–113]	97 [81–111]	0·58
Prehospital vasopressor use	71 (14·5%)	247 (14·4%)	0·60
Red flag alert ≥2	71 (14·5%)	251 (14·7%)	0·22
Initial capillary hemoglobin (g/dL)	13·0 [11·0–14·1]	12·9 [11·4–14·3]	0·19
Traumatic brain injury	133 (27·2%)	478 (28·0%)	0·29
Pupillary abnormality Anisocoria	66 (13·4%)	119 (6·9%)	<0·01
Mydriasis	25 (5·1%)	64 (3·7%)	<0·01
ICP monitoring	28 (5·7%)	139 (8·4%)	0·06
Neurosurgery	33 (6·7%)	90 (5·3%)	0·05
Significant bleeding	64 (13·0%)	179 (10·4%)	0·05
Platelet count (G/L)	204 [157–250]	212 [169–260]	<0·01
PT ratio >1·2	294 (60·1%)	410 (24·0%)	<0·01
Fibrinogen <1·5 g/L	24 (4·9%)	172 (10·5%)	<0·01
Outcome			
Length of hospital stay (days)	9·0 [3·0–21·0]	9·0 [3·0–22·0]	0·73
Length of ICU stay (days)	3·0 [2·0–7·0]	3·0 [2·0–8·0]	0·14
1-Day mortality	75 (15·1%)	178 (10·4%)	<0·01
7-Day mortality	133 (27·2%)	341 (19·9%)	<0·01
ICU mortality	165 (33·7%)	442 (25·8%)	<0·01

ASA score, American Society of Anesthesiologists score; BMI, Body Mass Index; TRISS, Trauma and Injury Severity Score; ISS, Injury Severity Score; GCS, Glasgow Coma Scale; MAP, Mean Arterial Pressure; ICP, Intracranial Pressure; PT, Prothrombin Time; ICU, Intensive Care Unit.

Both groups exhibited similar initial trauma characteristics including GCS and shock index. They required comparable prehospital management, such as tracheal intubation and vasopressor use, and had similar predicted probabilities of survival (based on the TRISS score) and of experiencing severe haemorrhage (based on the prehospital Red Flag alert^11^). However, a higher proportion of OAC-treated patients experienced significant bleeding (13·0% vs 10·4%, p = 0·05) and required RBC and FFP transfusion within the first 6 and 24 h (Figure S3A). VKA-treated patients also received more RBC (4 [2, 26] vs 3 [1, 23], p = 0·04) within the first 24 h than non-OAC-treated patients, but not DOAC-treated patients (4 [1, 24] vs 3 [1, 23], p = 0·39). Both VKA- and DOAC-treated groups also received more FFP (4 [1, 32] and 4 [2, 36] vs 3 [2, 19], p = 0·03 and p = 0·04 respectively). The amount of transfused platelets did not differ between OAC and non-OAC treated patients (Figure S3B). The proportion of patients with TBI was similar between OAC-treated and non-OAC-treated patients, as was the initial GCS score. However, pupillary abnormalities including anisocoria and bilateral mydriasis were more common among OAC-treated patients, as was the need for neurosurgery (Table 2).

The main reported causes of death were traumatic brain injury, withdrawal of life-sustaining therapy, sepsis and multiple organ failure, and haemorrhagic shock, and the proportions differed between OAC- and non-OAC-treated patients (28·8%, 36·2%, 20·2% and 3·7% vs 37·5%, 31%, 16·8% and 3·6% respectively, p = 0·35). On univariate analysis, pre-injury OAC therapy was associated with an increased risk of mortality at day 1 (OR = 1·89, 95% CI: 1·34–3·01, p = 0·01) and day 7 (OR = 1·67, 95% CI: 1·18–2·25, p < 0·01) (Tables S1 and S2). After adjustment for confounding factors, multivariate logistic regression analysis showed that OAC therapy was an independent risk factor for mortality at day 1 (OR = 2·21, 95% CI: 1·41–3·43, p < 0·01) and day 7 (OR = 2·06, 95% CI: 1·41–3·00, p < 0·01).

The same analysis was repeated, this time stratifying OAC therapy into VKA and DOAC use (Tables S3 and S4). Multivariate logistic regression analysis showed that pre-injury VKA therapy was an independent risk factor for mortality at day 1 compared with patients without OAC (OR = 2·41, 95% CI: 1·67–4·31, p < 0·01), as was pre-injury DOAC therapy (OR = 1·52, 95% CI: 1·09–2·88, p = 0·03). Similarly, both VKA therapy and DOAC therapy were independent risk factors for mortality at day 7 compared with patients without OAC in multivariate logistic regression (OR = 2·66, 95% CI: 1·89–3·99, p < 0·001 and OR = 1·84, 95% CI: 1·21–3·17, p = 0·02 respectively).

### Anticoagulant reversal and mortality

**Fig. 2:**
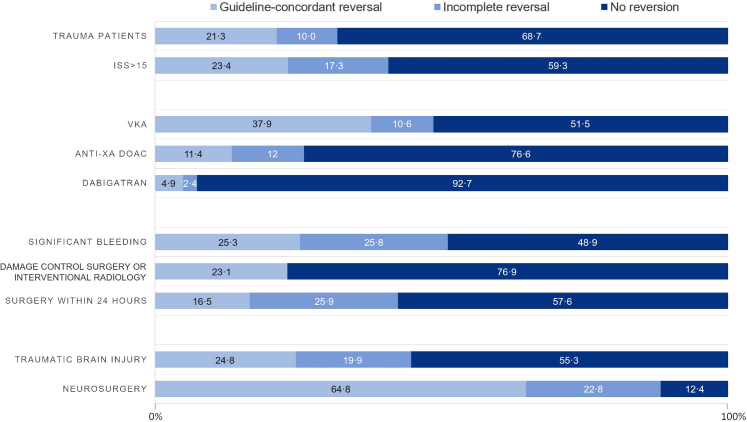
*Rates (%) of OAC reversal in OAC-treated patients according to the anticoagulant and trauma.* For example, the bottom band indicates that among OAC-treated patients undergoing neurosurgery 64·8% had guideline-concordant reversal and 12·4% had no reversal. Anti-Xa DOAC, Direct Oral Anticoagulant targeting Factor Xa; ISS, Injury Severity Score; VKA, Vitamin K Antagonist.

Of the 489 OAC-treated patients, 69 (14·1%) received PCCs within the first 24 h, with a median dose of 31 [23–43] IU/kg. No patients received idarucizumab, andexanet alpha or rFVIIa. Only 21·3% (n = 104) received guideline-concordant reversal, 10·0% (n = 49) had incomplete reversal, and 68·7% (n = 336) had no reversal. The use of reversal varied according to the characteristics of the anticoagulant and the trauma (Fig. 2): guideline-concordant reversal was more frequently performed in patients on VKAs than in those on DOACs (37·9% [69/182] vs 11·4% [35/307], p < 0·01). It was more frequent in severe trauma patients (ISS >15) compared to non-severe ones (23·4% [49/209] vs 17·0% [34/206], p < 0·01), in patients with significant bleeding (25·3% [16/63] vs 16·4% [70/426], p = 0·03), and in those with TBI (24·8% [36/145] vs 16·9% [50/296], p = 0·01). It was much more frequently performed in patients undergoing neurosurgery, compared to those requiring damage control surgery or interventional radiology and those with significant bleeding (64·8%; 23·1% and 25·3% respectively, p < 0·01).

**Fig. 3:**
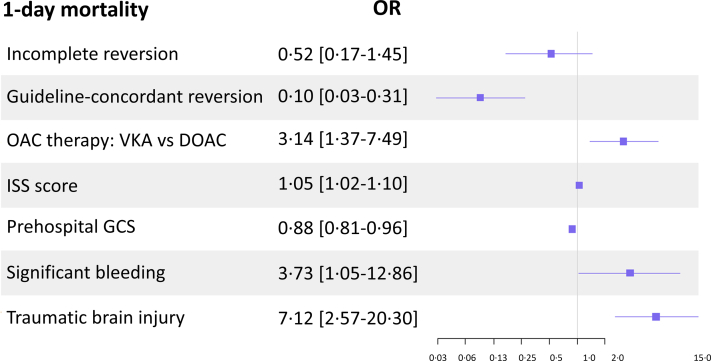
*Multivariate logistic regression analysis of 1-day mortality in OAC-treated patients.* GCS, Glasgow Coma Scale; DOAC, Direct Oral Anticoagulant; ISS, Injury Severity Score; OAC, Oral Anticoagulant; VKA, Vitamin K Antagonist.

In OAC-treated patients, multivariate logistic regression analysis showed that guideline-concordant OAC reversal was an independent protective factor against 1-day mortality (OR 0·10, 95% CI [0·03–0·31], p < 0·01) whereas incomplete reversal did not reduce mortality (Fig. 3 and Table S5). Other independent risk factors for 1-day mortality were VKA therapy compared to DOACs (OR 3·14, 95% CI [1·37–7·49], p = 0·01), ISS, GCS, TBI and significant bleeding. Similarly, guideline-concordant OAC reversal also reduced 7-day mortality (OR 0·51, 95% CI [0·22–0·97]), but, again, incomplete reversal did not (OR 0·45, 95% CI [0·17–1·21]).

**Fig. 4:**
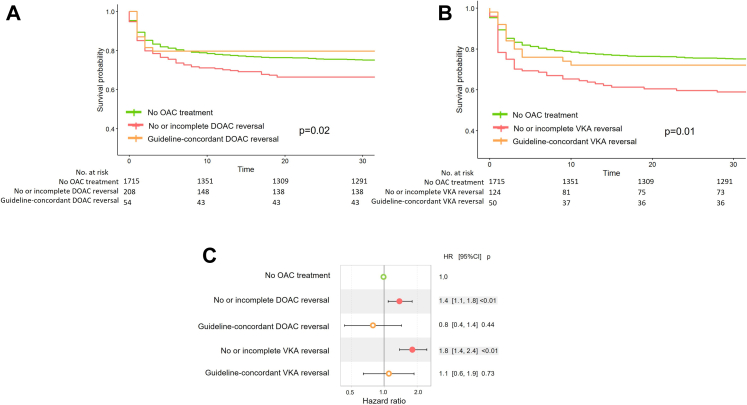
***A, B, C:****Association between quality of reversal and mortality according to anticoagulant agent using survival analysis with Cox regression in the ARIANE cohort.* OAC, Oral Anticoagulant; DOAC, Direct Oral Anticoagulant; VKA, Vitamin K Antagonist.

The benefit of reversal was observed regardless of the anticoagulant agent (Fig. 4): VKA-treated patients with no or incomplete reversal had increased mortality compared with non-OAC-treated patients (HR 1·8 95% CI [1·4–2·4], p < 0·01) whereas after guideline-concordant reversal, VKA-treated patients had a mortality rate similar to that of non-OAC-treated patients (HR 1·1 95% CI [0·6–1·9], p = 0·73). Same results were observed in DOAC-treated patients: those with no or incomplete reversal had increased mortality compared with non-OAC-treated patients (HR 1·4 95% CI [1·1–1·8], p < 0·01), whereas after guideline-concordant reversal, DOAC-treated patients had a mortality rate similar to that of non-OAC-treated patients (HR 1·1 95% CI [0·4–1·4], p = 0·44).

### Resumption of anticoagulation

Anticoagulant prophylaxis was prescribed for 70·7% of the 256 pre-injury OAC-treated patients who were hospitalised in the ICU for more than 48 h, with initiation occurring within 2·0 [1·0, 4·0] days of admission. Therapeutic anticoagulation was reintroduced in the ICU for only 54 (29·9%) patients, primarily using LMWH (64·8%) or UFH (32·2%). The reintroduction time was 5·5 [2·3, 7·8] days. Patients with a mechanical heart valve were more likely to resume therapeutic anticoagulation in the ICU (60% (6/10) vs 27·9% (48/172), p = 0·03). Resumption of therapeutic anticoagulation was associated with bleeding complications in five patients (9·2%), requiring blood transfusion (n = 3), embolisation (n = 2) or surgery (n = 2).

**Fig. 5:**
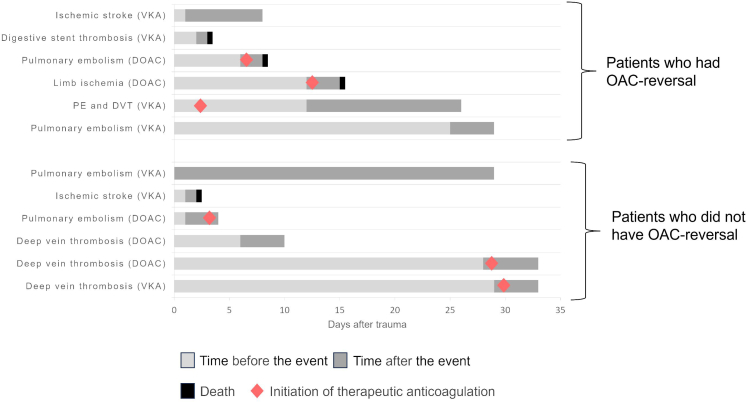
*Thrombotic Events in ICU according to anticoagulant reversal in trauma patients with preinjury OAC therapy.* Among the 257 pre-injury OAC-treated patients who were hospitalised in the ICU for more than 48 h, 12 (4·7%) patients experienced a thrombotic event in the ICU. The figure described these events: the type of pre-injury anticoagulant agent is listed in brackets after the type of thrombotic event. The light gray portion of the bar indicates the time from hospital admission to the thrombotic event, the dark gray portion indicates the time in the ICU after the event and the black portion indicates death. Red diamonds indicate the initiation of therapeutic anticoagulation. PE, pulmonary embolism; DVP, deep vein thrombosis.

Among the 256 OAC-treated patients, 12 (4·7%) patients experienced a thrombotic event in the ICU and four of them died (Fig. 5). The association between thrombotic events and anticoagulant reversal was not statistically significant (1·1% for no or incomplete reversal, 5·7% for guideline-concordant reversal, p = 0·09). Thrombotic events were not significantly associated with the type of anticoagulant used (58·3% with VKA, 41·7% with DOAC, p = 0·59) All but one occurred before therapeutic anticoagulation was resumed.

## Discussion

Patients on preinjury anticoagulants accounted for 3% of all trauma patients of our registry, a proportion similar to that observed in other cohorts where 3–6% of trauma patients had anticoagulant treatment.^12^^,^^13^ With the rising use of DOACs,^14^ a corresponding increase in DOAC-treated trauma patients is expected, as observed in our study. OAC-treated trauma patients have higher mortality rates than other trauma patients.^15^^,^^16^ However, they are generally older and have more comorbidities, which also contribute to an increased risk of poor outcomes. In the ARIANE cohort, after matching for age, sex, trauma type and year of injury —and despite comparable pre-hospital characteristics and management, and scores of injury severity and mortality prediction— OAC-treated patients exhibited higher mortality than non-OAC-treated patients. Our analysis confirms previous studies showing that VKAs increase mortality more than DOACs in similar populations of patients with moderate to severe trauma,^17^ mild trauma,^18^ severe blunt trauma^19^ and TBI.^20^ Our analysis also concluded that DOACs increase mortality compared to non-OAC-treated patients, whereas previous studies have reported conflicting results, which may be due to small sample sizes and an inability to fully account for residual confounders such as comorbidities.^16^^,^^17^^,^^21^, ^22^, ^23^, ^24^ Nevertheless, these findings provide reassurance about the overall safety of DOACs and highlight their advantage over VKAs in bleeding patients.

Reversal strategies depend on the anticoagulant agent. For VKA reversal, PCCs replace the missing coagulation factors, FII, FVII, FIX and FX, restoring haemostasis and immediately normalizing the INR. For DOAC reversal, two specific antidotes are marketed: idarucizumab binds dabigatran with high affinity and specificity, reducing circulating unbound dabigatran, while andexanet alpha binds and sequesters FXa inhibitors, including apixaban, rivaroxaban and edoxaban, reducing anti-FXa activity. If these antidotes are unavailable—or before they were marketed—PCCs serve as a non-specific reversal option. These agents are usually proposed to reverse anticoagulants in bleeding patients^1^^,^^5^^,^^6^^,^^25^ although their benefit on robust clinical endpoints remains unproven. Studies supporting their use often focus on surrogate markers, such as laboratory indicators of coagulation improvement including INR for VKA^26^ and unbound DOAC concentrations,^23^^,^^24^ or radiographic evidence of intracranial hemorrhage progression.^25^^,^^26^ The clinical benefit of reversal strategies is primarily supported by observational studies: the analysis of 822 VKA-treated patients with severe hemorrhage (gastrointestinal 32% intracranial 32%) reported that guideline-concordant VKA reversal with PCC and vitamin K was associated with a significant decrease in 7-day mortality.^27^ Similarly, the retrospective study of 1771 VKA-treated patients with severe hemorrhage from various sites showed that INR correction was associated with better survival.^28^ Mortality reduction associated with reversal was also observed in both cohorts when only intracranial haemorrhage was considered. In RCT including VKA- or anti-Xa DOAC-treated patients with intracranial haemorrhage, reversal resulted in smaller hematoma expansion compared to control, but none demonstrated a decreased mortality.^25^^,^^26^ Singh et al. even reported increased in-hospital mortality associated with reversal in a retrospective cohort study comparing idarucizumab vs control in 112 patients with dabigatran-related intracranial haemorrhage, although the study had several limitations.^29^ In trauma patients, there is very limited evidence regarding the impact of anticoagulant reversal. As others,^7^^,^^9^ we observed that reversal is underused and proposed to the most severe patients including those requiring neurosurgery, as their high bleeding risk necessitates optimal haemostasis. We showed for the first time that guideline-concordant OAC-reversal was an independent protective factor of trauma mortality. More widespread use of specific antidotes such as andexanet alfa and idarucizumab could increase the clinical benefit of reversal. However, their cost and limited availability in certain centres and countries are obstacles to their use. These data strengthen the recommendations and confirm that more efforts are needed to comply with them, despite the challenges in the emergency setting. The implementation of pragmatic local protocols defining the role of biology, the use of antidotes or CCPs, and individual dosages would increase adherence and improve the prognosis for trauma patients.

We reported 4·7% of thrombotic events in the ICU, which is close to the 5–10% reported in studies evaluating anticoagulant reversal,^26^^,^^30^, ^31^, ^32^, ^33^ where these complications were closely monitored. The benefits of anticoagulant reversal must be weighed against the potential harms, particularly the prothrombotic effects of reversal agents that combined with trauma-associated thrombotic risk, prothrombotic inflammatory responses to bleeding, patient's intrinsic pre-existing prothrombotic risk and anticoagulation discontinuation. Andexanet significantly increases thrombotic events compared with PCC, including ischemic stroke.^33^ In contrast, RCTs evaluating PCC for VKA reversal did not report a higher prevalence of thrombotic events compared to controls.^26^^,^^32^ Nevertheless, in a RCT including trauma patients at risk of massive transfusion but not receiving preinjury anticoagulation, PCC increased thrombotic events compared with placebo.^34^ Although not statistically significant, the absolute difference in thrombotic events observed in our study (5·7% after guideline-concordant reversal vs 1·7% without reversal) raises concerns and emphasises the need for vigilance and prompt reinitiation of anticoagulation when feasible.

There is uncertainty about whether and when to restart anticoagulants after bleeding: resuming too early increases the risk of rebleeding, while delaying too long increases the risk of thrombotic events. In our cohort, anticoagulant resumption occurred in the ICU in fewer than one in five patients, most frequently in those with a mechanical heart valve. Similarly, in other cohorts studying anticoagulant reversal for bleeding, anticoagulants were restarted in only 20%–28% of patients.^9^^,^^31^ In our study, resumption occurred early, mostly within the first week, and was associated with bleeding complications in 9% of cases, consistent with the 4·5% bleeding event rate reported in the ANNEXA-4 cohort after anticoagulant resumption.^35^ All but one of the thrombotic events occurred before therapeutic anticoagulation was resumed, highlighting the difficulty of individualizing the optimal time for resumption. A post-hoc analysis of ANNEXA-4 suggested that in patients with DOAC-associated bleeding, restarting anticoagulation was associated with an increased risk of rebleeding but a reduced risk of thrombotic events, resulting in a potential net benefit for restarting.^35^

This study has several limitations. First, as a retrospective analysis, it limits the ability to account for specific confounders, such as local management strategies or dynamic clinical responses to treatment. Nevertheless, data collection was primarily prospective and the use of the Traumabase® registry secures data processing, minimized missing data through responsive data-monitoring. It was not possible to randomise preinjury OAC therapy, so matching was rigorous to reduce the number of confounders. The long study period (2012–2023) may have introduced bias, as DOACs were marketed in 2012 and specific antidotes remained scarcely available. To address this, VKAs and DOACs were analysed separately, and the year of trauma was included as a covariate in the propensity score analysis. More extensive matching criteria could have been applied, but doing so would have reduced statistical power. Although the propensity score matching was performed using a 1:4 nearest-neighbour algorithm without replacement, it was not possible to match all OAC-treated patients all OAC-treated patients to four controls due to the constraints of the matching algorithm and the limited number of suitable control matches within the caliper. Nevertheless, matching according to the selected criteria resulted in comparable initial trauma characteristics, suggesting that we reduced bias and provided relevant data on OAC-associated mortality. The logistic regression model that assessed the relationship between reversal and mortality was also strictly performed to control for confounding. However, the sample size was limited —particularly in the subgroup of patients who received incomplete reversal— so some of the non-significant results might be due to the lack of power. It was clinically important to distinguish this group from both patients receiving guideline-concordant reversal and those with no reversal at all, given the potential implications of underdosing or partial reversal. Although the cause of death usually differs between trauma patients with and without TBI, there were too few OAC-treated patients to analyse them separately. Therefore, the benefit of reversal may vary according to TBI. Second, survival bias cannot be entirely ruled out, especially for traumatic brain injury, as was the opposite bias leading to reverse OAC in patients with more severe injuries. Third, non-compliance with preinjury OAC treatment may have affected the effects of OAC and the impact of reversal on mortality. Although data on anticoagulant level at admission were available for only 60% of patients, non-compliance was observed in only 20% of both DOAC- and VKA-treated patients, which limited bias. Nevertheless, if patients who were not truly anticoagulated at the time of trauma could have been identified and excluded, the observed association between reversal and mortality might have been even more robust. Moreover, adequacy of VKA reversal is usually measured by post-PCC INR results, although the relationship between post-PCC INR and vitamin K-dependent factor levels is uncertain, which may have over- or underestimate effectiveness.^36^ As this data was not available, the amount of PCC—median dose of 30 UI/kg, similar to that used in RCTs assessing VKA reversal^26^^,^^32^–and the guideline concordance were used as surrogate markers of adequate reversal. Fourth, the low incidence of thrombotic events suggests potential underreporting. Last, our study did not catch the impact of preexisting frailty or loss of autonomy in this older-than-usual population, limiting our ability to assess their association with anticoagulant management and poor outcomes.

In conclusion, OAC-treated patients accounted for 3% of the trauma cohort, were older, had more comorbidities and experienced higher mortality. After adjustment, both VKA and DOACs increased mortality and PCC-based guideline-concordant reversal emerged as an independent protective factor without significant rise in thrombotic events. Further studies are needed to determine the optimal PCC dosage and the advantages of specific DOAC antidotes.

## Contributors

EL, MG, JLH, FV, GA, DG, PSA, JP, and AG conceived and designed the study.

All authors actively participated in data collection.

EL, MG and AG accessed and verified the underlying data.

EL performed the statistical analysis.

EL, MG, FV, DG, and AG drafted the manuscript.

AG was responsible for the decision to submit the manuscript.

All authors and collaborators critically revised the manuscript for important intellectual content and approved the final version.

## Data sharing statement

Individual participant data from the Traumabase® registry and the ARIANE cohort cannot be shared publicly because of French data protection regulations and approvals from the French National Commission on Computing and Liberty (CNIL).

Aggregated, anonymized data underlying the findings of this study may be made available upon reasonable request to the corresponding author. Any data sharing will require a detailed proposal, review, and approval by the Traumabase scientific committee and relevant ethics committees, in compliance with applicable legal and regulatory frameworks.

## Declaration of interests

Elodie Lang, Marion Gautier, Fanny Vardon, Vincent Legros, Jean-Luc Hanouz, Florent Hericher, Olivier Duranteau, Pierre-Antoine Allain, Benjamin Popoff, Thomas Botrel, and Jean Pasqueron declare no competing interests.

Anne Godier reports personal fees from Aguettant, Alexion, Bayer Healthcare, BMS-Pfizer, Boehringer Ingelheim, Sanofi, CSL Behring, LFB, Octapharma, Stago, and Viatris.

Delphine Garrigue Huet reports personal fees from LFB, Octapharma, Chugai, Boehringer Ingelheim, Bayer, and AstraZeneca.

Gérard Audibert reports personal fees from LFB and Octapharma.

Gary Duclos reports lecture fees from AOP Health.

Paer-Sélim Abback reports personal fees from LFB.

Samy Figueiredo reports personal fees from Edwards Lifesciences and Octapharma.

## Appendix A Supplementary data

Supplementary Data

Ethics Approval
