# Supplementary material for: Oral anticoagulant reversal and mortality in trauma patients: a multicentre propensity score–matched cohort study
Source: eClinicalMedicine. 2025 Oct 16;89:103577. doi: 10.1016/j.eclinm.2025.103577 (PMC12554126; doi:10.1016/j.eclinm.2025.103577)
Supplement: Supplementary Data [file mmc1.docx]

**Supplementary data**

[Figure S1: Standardized mean differences of covariates before and after propensity score matching and model discrimination for the propensity score. 2](#_Toc207185472)

[Figure S2: Proportion of patients treated with oral anticoagulants. 2](#_Toc207185473)

[Figure S3: Transfusion at 6 hours and 24 hours after admission according to OAC therapy in trauma patients in the ARIANE cohort. 3](#_Toc207185474)

[A/ Rate of Transfusion 3](#_Toc207185475)

[B/ Amount of transfused blood products 3](#_Toc207185476)

[Table S1: Multivariate logistic regression analysis of factors associated with 1-day mortality in the ARIANE cohort. 4](#_Toc207185477)

[Table S2: Multivariate logistic regression analysis of factors associated with 7-day mortality in the ARIANE cohort. 5](#_Toc207185478)

[Table S3: Multivariate logistic regression analysis of factors associated with 1-day mortality in the ARIANE cohort. 6](#_Toc207185479)

[Table S5: Multivariate logistic regression analysis of 1-day mortality in OAC-treated patients. 8](#_Toc207185480)

# Figure S1: Standardized mean differences of covariates before and after propensity score matching and model discrimination for the propensity score.


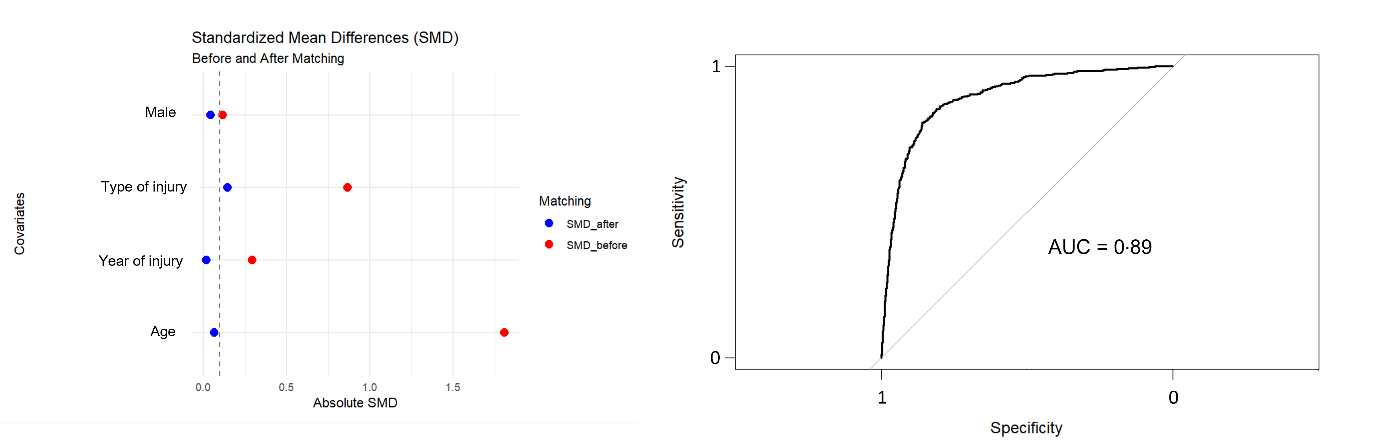


*AUC: area under the curve ; SMD: standardized mean difference*

# Figure S2: Proportion of patients treated with oral anticoagulants.


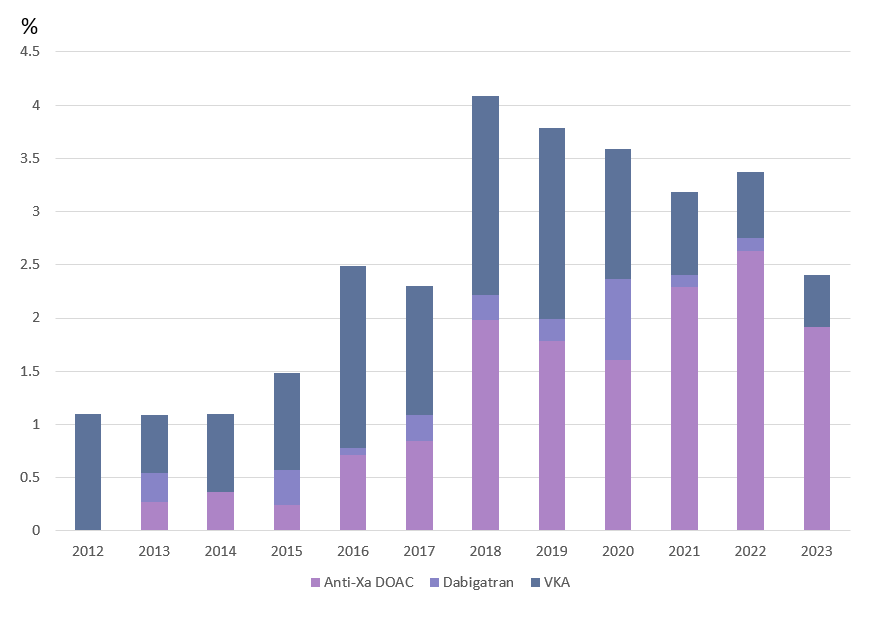


DOAC: Direct Oral Anticoagulant ; OAC: Oral Anticoagulant; VKA: Vitamin K Antagonist.

# Figure S3: Transfusion at 6 hours and 24 hours after admission according to OAC therapy in trauma patients in the ARIANE cohort.

# A/ Rate of Transfusion

# B/ Amount of transfused blood products


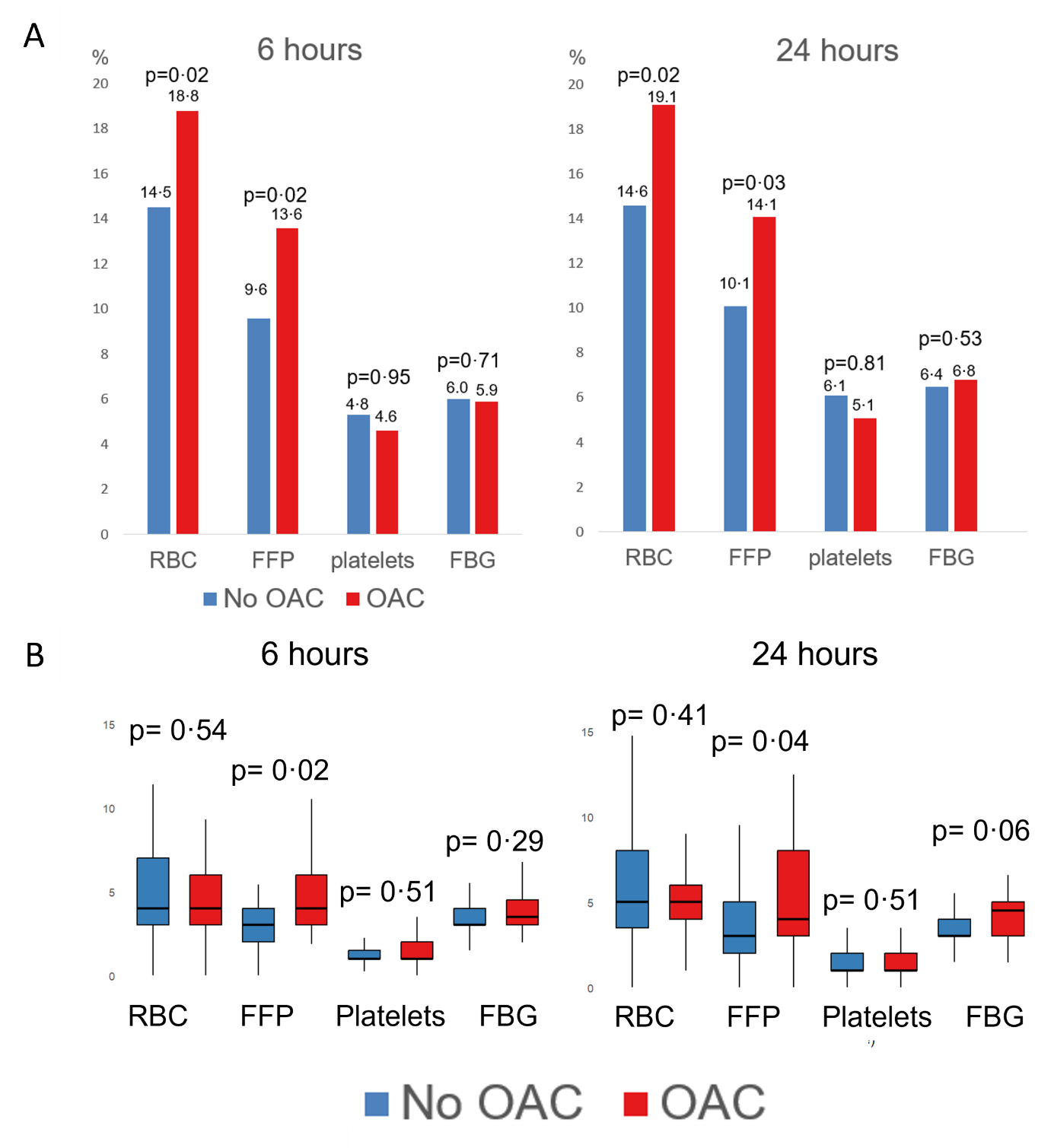


FBG: fibrinogen concentrates (g); FFP: Fresh Frozen Plasma Unit ; OAC: Oral Anticoagulant ; Platelets: Platelet unit; RBC: Red Blood Cell unit

We usually considered that one platelet concentrate was equivalent to five donor Platelet units (Hamada et al. Critical Care 2022).

# Table S1: Multivariate logistic regression analysis of factors associated with 1-day mortality in the ARIANE cohort.

| **1-Day Mortality** | **Univariate analyse** | | **Multivariate analyse** | |
| --- | --- | --- | --- | --- |
|  | **Odds-Ratio (95% CI, p)** |  | **Odds-Ratio (95% CI, p)** |  |
| OAC therapy vs no OAC therapy | 1·89 (1·34-3·01, p=0·01) |  | 2·21 (1·41-3·43, p<0·01) |  |
| Age | 1·04 (1·03-1·05, p<0·01) |  | 1·04 (1·03-1·06, p<0·01) |  |
| Male | 0·93 (0·69-1·26, p=0·64) |  | ·· |  |
| BMI | 0·99 (0·96-1·02, p=0·65) |  | ·· |  |
| ISS score | 1·07 (1·06-1·08, p<0·01) |  | 1·03 (1·02-1·05, p<0·01) |  |
| Antiplatelet therapy | 0·45 (0·97-1·09, p=0·24) |  | ·· |  |
| Penetrating trauma | 1·10 (0·73-1·61, p=0·63) |  | ·· |  |
| Significant bleeding | 3·14 (2·26-4·33, p<0·01) |  | 1·96 (0·84-4·37, p=0·11) |  |
| Multiple sources of bleeding | 5·77 (2·57-12·49, p<0·001) |  | 2·51 (0·58-10·14, p=0·21) |  |
| Prehospital GCS | 0·80 (0·77-0·82, p<0·01) |  | 0·84 (0·80-0·88, p<0·01) |  |
| Red flag alert ≥2 | 2·06 (1·25-3·27, p=0·01) |  | 3·10 (0·99-6·58, p=0·10) |  |
| Damage control surgery or interventional radiology | 1·28 (0·65-2·31, p=0·44) |  | ·· |  |
| Traumatic brain injury | 7·31 (5·20-10·45, p<0·01) |  | 4·78 (2·31-6·44, p<0·01) |  |

BMI: Body Mass Index; GCS: Glasgow Coma Scale; ISS: Injury Severity Score; OAC: Oral Anticoagulant.

# Table S2: Multivariate logistic regression analysis of factors associated with 7-day mortality in the ARIANE cohort.

| **7-Day Mortality** | **Univariate analyse** | | **Multivariate analyse** | |
| --- | --- | --- | --- | --- |
|  | **Odds-Ratio (95% CI, p)** |  | **Odds-Ratio (95% CI, p)** |  |
| OAC therapy vs no OAC therapy | 1·67 (1·18-2·25, p<0·01) |  | 2·06 (1·41-3·00, p<0·01) |  |
| Age | 1·04 (1·04-1·05, p<0·01) |  | 1·05 (1·01-1·07, p<0·01) |  |
| Male | 0·96 (0·76-1·21, p=0·72) |  | ·· |  |
| BMI | 1·00 (0·97-1·02, p=0·72) |  | ·· |  |
| ISS score | 1·10 (1·08-1·11, p<0·01) |  | 1·06 (1·05-1·08, p<0·01) |  |
| Antiplatelet therapy | 1·62 (0·90-2·05, p=0·22) |  | ·· |  |
| Penetrating trauma | 0·99 (0·72-1·35, p=0·95) |  | ·· |  |
| Significant bleeding | 2·37 (1·78-3·14, p<0·01) |  | 2·31 (1·62-3·44, p<0·001) |  |
| Multiple sources of bleeding | 3·05 (1·39-6·55, p=0·042) |  | 0·73 (0·38-7·52, p=0·75) |  |
| Prehospital GCS | 0·77 (0·75-0·79, p<0·01) |  | 0·81 (0·78-0·84, p<0·001) |  |
| Red flag alert ≥2 | 140 (0·90-2·13, p=0·12) |  | 1·86 (0·88-3·77, p=0·09) |  |
| Damage control surgery or interventional radiology | 0·96 (0·55-1·61, p=0·89) |  | ·· |  |
| Traumatic brain injury | 8·05 (6·96-10·84, p<0·01) |  | 7·27 (2·32-21·17, p<0·001) |  |

BMI: Body Mass Index; GCS: Glasgow Coma Scale; ISS: Injury Severity Score; OAC: Oral Anticoagulant.

# Table S3: Multivariate logistic regression analysis of factors associated with 1-day mortality in the ARIANE cohort.

| **1-Day Mortality** | **Univariate analyse** | | **Multivariate analyse** | |
| --- | --- | --- | --- | --- |
|  | **Odds-Ratio (95% CI, p)** |  | **Odds-Ratio (95% CI, p)** |  |
| VKA therapy vs no OAC therapy | **1·64 (1·21-2·55, p=0·01)** |  | **2·41 (****1·67-4·31, p<0·01)** |  |
| DOAC therapy vs no OAC therapy | **1·41 (1·05-1·94, p=0·04)** |  | **1·52 (1·09-2·88, p=0·03)** |  |
| Age | 1·04 (1·03-1·05, p<0·01) |  | 1·03 (1·04-1·09, p<0·01) |  |
| Male | 0·93 (0·69-1·26, p=0·64) |  | ·· |  |
| BMI | 0·99 (0·96-1·02, p=0·65) |  | ·· |  |
| ISS score | 1·07 (1·06-1·08, p<0·01) |  | 1·08 (1·04-1·12, p<0·01) |  |
| Antiplatelet therapy | 0·45 (0·97-1·09, p=0·24) |  | ·· |  |
| Penetrating trauma | 1·10 (0·73-1·61, p=0·63) |  | ·· |  |
| Significant bleeding | 3·14 (2·26-4·33, p<0·01) |  | 3·92 (1·04-8·52, p=0·04) |  |
| Multiple sources of bleeding | 5·77 (2·57-12·49, p<0·001) |  | 2·64 (0·59-10·42, p=0·45) |  |
| Prehospital GCS | 0·80 (0·77-0·82, p<0·01) |  | 0·92 (0·88-0·97, p<0·01) |  |
| Red flag alert ≥2 | 2·06 (1·25-3·27, p=0·01) |  | 3·21 (0·51-6·56, p=0·67) |  |
| Damage control surgery or interventional radiology | 1·28 (0·65-2·31, p=0·44) |  | ·· |  |
| Traumatic brain injury | 7·31 (5·20-10·45, p<0·01) |  | 4·02 (2·32-6·51, p=0·04) |  |

BMI: Body Mass Index; DOAC: Direct Oral Anticoagulant; GCS: Glasgow Coma Scale; ISS: Injury Severity Score; OAC: Oral Anticoagulant; VKA: Vitamin K Antagonist.

**Table S4: Multivariate logistic regression analysis of factors associated with 7-day mortality in the ARIANE cohort.**

| **7-Day Mortality** | **Univariate analyse** | | **Multivariate analyse** | |
| --- | --- | --- | --- | --- |
|  | **Odds-Ratio (95% CI, p)** |  | **Odds-Ratio (95% CI, p)** |  |
| VKA therapy vs no OAC therapy | 1·45 (1·12-2·25, p<0·01) |  | **2·66 (1·89-3·99, p<0·01)** |  |
| DOAC therapy vs no OAC therapy | 1·37 (1·08-2·44, p=0·04) |  | **1·84 (1·21-3·17, p=0·02)** |  |
| Age | 1·04 (1·04-1·05, p<0·01) |  | 1·07 (1·02-1·10, p<0·01) |  |
| Male | 0·96 (0·76-1·21, p=0·72) |  | ·· |  |
| BMI | 1·00 (0·97-1·02, p=0·72) |  | ·· |  |
| ISS score | 1·10 (1·08-1·11, p<0·01) |  | 1·06 (1·05-1·08, p<0·001) |  |
| Antiplatelet therapy | 1·62 (0·90-2·05, p=0·22) |  | ·· |  |
| Penetrating trauma | 0·99 (0·72-1·35, p=0·95) |  | ·· |  |
| Major bleeding | 2·37 (1·78-3·14, p<0·01) |  | 2·28 (1·69-3·51, p<0·01) |  |
| Multiple sources of bleeding | 3·05 (1·39-6·55, p=0·042) |  | 0·81 (0·31-7·42, p=0·59) |  |
| Prehospital GCS | 0·77 (0·75-0·79, p<0·01) |  | 0·81 (0·78-0·84, p<0·001) |  |
| Red flag alert ≥2 | 140 (0·90-2·13, p=0·12) |  | 1·89 (0·84-3·96, p=0·64) |  |
| Damage control surgery or interventional radiology | 0·96 (0·55-1·61, p=0·89) |  | ·· |  |
| Traumatic brain injury | 8·05 (6·96-10·84, p<0·01) |  | 7·53 (2·45-22·37, p<0·01) |  |

BMI: Body Mass Index; DOAC: Direct Oral Anticoagulant; GCS: Glasgow Coma Scale; ISS: Injury Severity Score; OAC: Oral Anticoagulant; VKA: Vitamin K Antagonist.

# Table S5: Multivariate logistic regression analysis of 1-day mortality in OAC-treated patients.

| **1-day mortality** | **Univariate analyse** | | **Multivariate analyse** | |
| --- | --- | --- | --- | --- |
|  | **Odds-Ratio [95% CI]** | ***p*** | **Odds-Ratio [95% CI]** | ***p*** |
| Incomplete reversion vs no reversion | 1·60 [0·84-2·98] | 0·15 | 0·52 [0·17-1·45] | 0·22 |
| Guideline-concordant reversion vs no reversion | **0·52 [0·24-1·54]** | **0·06** | **0·10 [0·03-0·31]** | **< 0·01** |
| OAC therapy: VKA vs DOAC | **1·91 [1·26-2·90]** | **< 0·01** | **3·14 [1·37-7·49]** | **0·01** |
| Age | 1·02 [1·01-1·04] | 0·01 | ·· | ·· |
| Male | 0·89 [0·58-1·38] | 0·60 | ·· | ·· |
| ISS score | 1·11 [1·08-1·14] | < 0·01 | 1·05 [1·02-1·10] | 0·01 |
| Significant bleeding | 1·76 [1·01-3·02] | 0·04 | 3·73 [1·05-12·86] | 0·04 |
| Multiple sources of bleeding | 0·66 [0·03-4·50] | 0·71 | ·· | ·· |
| Prehospital GCS | 0·78 [0·75-0·82] | < 0·01 | 0·88 [0·81-0·96] | 0·01 |
| Prehospital intubation | 0·77 [0·48-6·13] | 0·55 | ·· | ·· |
| Red flag alert ≥ 2 | 1·62 [0·77-3·29] | 0·21 | ·· | ·· |
| *RBC transfusion within 6h* | 1·05 [0·96-1·15] | 0·26 | ·· | ·· |
| *FFP transfusion within 6h* | 1·01 [0·87-1·16] | 0·86 | ·· | ·· |
| *Platelet transfusion within 6h* | 0·99 [0·57-1·48] | 0·94 | ·· | ·· |
| *FBG administration within 6h* | 0·75 [0·51-1·02] | 0·09 | ·· | ·· |
| *Damage control surgery or interventional radiology* | 0·19 [0·01-1·12] | 0·22 | ·· | ·· |
| *Traumatic brain injury* | 11·97 [7·29-20·18] | < 0·01 | 7·12 [2·57-20·30] | < 0·01 |

DOAC: Direct Oral Anticoagulant; FBG: Fibrinogen; FFP: Fresh Frozen Plasma; GCS: Glasgow Coma Scale; ISS: Injury Severity Score; OAC: Oral Anticoagulant; RBC: Packed Red Blood Cells; VKA: Vitamin K Antagonist.
