# Supplementary material for: Oral anticoagulant reversal and mortality in trauma patients: a multicentre propensity score–matched cohort study
Source: eClinicalMedicine. 2025 Oct 16;89:103577. doi: 10.1016/j.eclinm.2025.103577 (PMC12554126; doi:10.1016/j.eclinm.2025.103577)

**Ethical approval**

The Traumabase® is in accordance with all requirements from the Advisory Committee for the processing of research information in the field of health (CCTIRS), the French National Commission on Computing and Liberty (CNIL, authorization number 911461) and meets the requirements of the local and national ethics committee (Comité de Protection des Personnes, Paris VI). The current study received a complementary ethical committee approval (CERAR – IRB 00010254 – 2025 - 044).


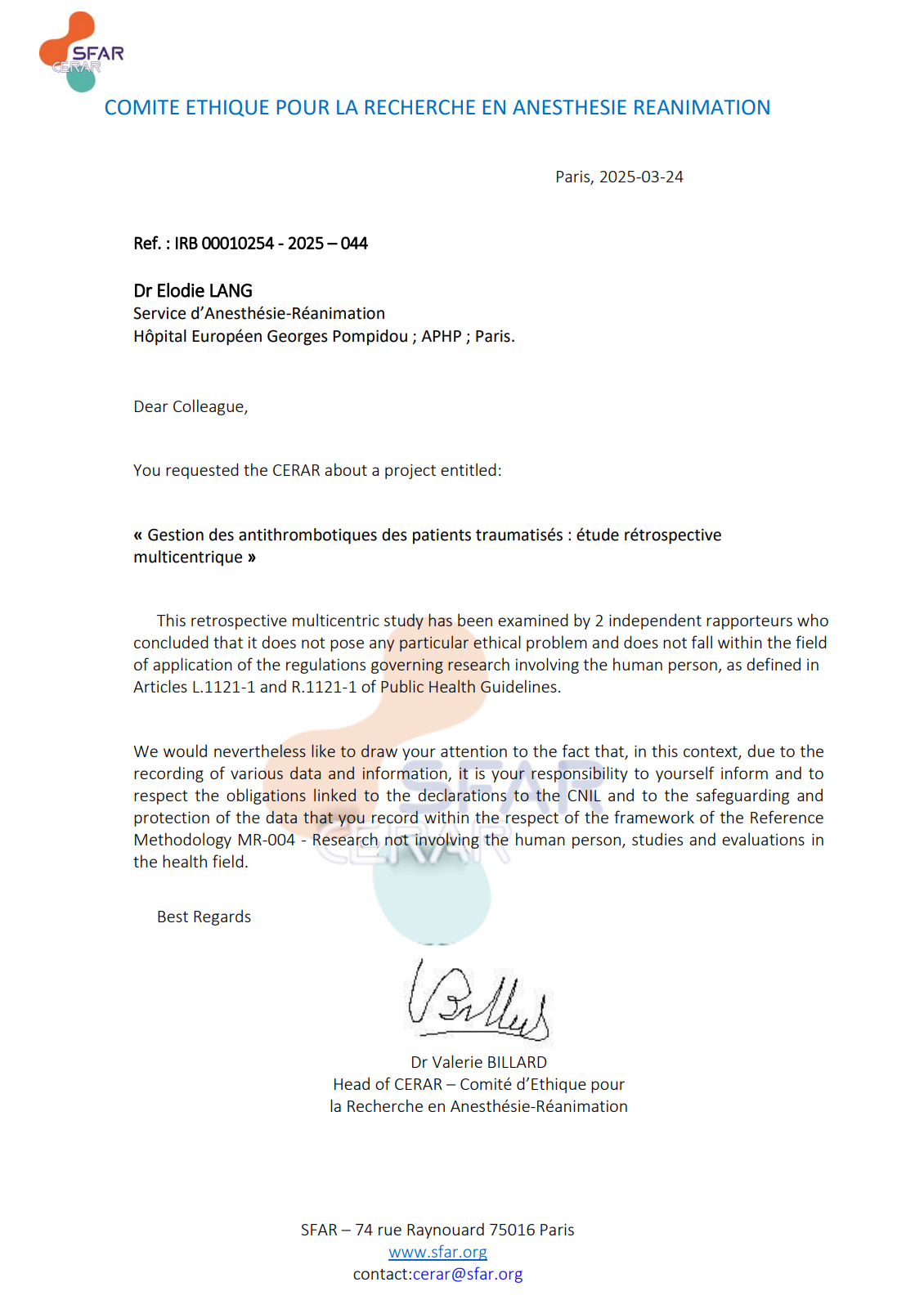

Supplement: Ethics Approval [file mmc2.docx]
